# Prognostic factors of lung abscess: a single-center retrospective cohort study

**DOI:** 10.1186/s12890-025-04002-3

**Published:** 2025-11-11

**Authors:** Hiroki Kawakami, Hiroki Matsui, Soichiro Yamaji, Norihiko Kubota, Tatsuya Nagai, Ayumu Otsuki, Hiroyuki Ito, Kei Nakashima

**Affiliations:** 1 Department of Pulmonology, Kameda Medical Center, 929 Higashi-cho, Kamogawa, Chiba 296-8602 Japan; 2 Clinical Research Support Office, Kameda Medical Center, Chiba, Japan; 3 Department of Clinical Epidemiology and Health Economics, School of Public Health, The University of Tokyo, Tokyo, Japan

**Keywords:** Lung abscess, Multiloculation, Diameter, Prognostic factors

## Abstract

**Background:**

A lung abscess, a localized suppurative necrosis of the lung parenchyma, remains a serious condition despite modern antibiotic therapy, with many patients requiring invasive procedures due to treatment failure. Identifying these high-risk patients is crucial, but robust prognostic factors are not well-established due to methodological limitations in prior studies, such as reliance on univariate analyses or heterogeneous endpoints. We, therefore, conducted a 20-year retrospective study to identify predictors for a composite outcome of treatment failure, defined as in-hospital death, drainage, or surgery.

**Methods:**

This single-center, retrospective cohort study included patients with lung abscesses who were hospitalized and initially managed with conservative antibiotic therapy at the Department of Pulmonology, Kameda Medical Center, between April 2004 and June 2024. The primary endpoint was treatment failure, defined as a composite of computed tomography-guided percutaneous catheter drainage of the lung abscess, surgery, and in-hospital death. Adjusted odds ratios (aORs) and 95% confidence intervals (CIs) for treatment failure were calculated using multivariate logistic regression analysis, adjusting for potential predictive variables, such as age, sex, comorbidity, and abscess characteristics, based on previous studies.

**Results:**

This study included 109 patients (mean age: 74 years), of whom 21 (19.3%) were female. Treatment failure was observed in 17 (15.6%) patients, including 9 (8.3%) in-hospital deaths, 1 (0.9%) surgical intervention, and 11 (10.1%) cases requiring CT-guided drainage of the lung abscess. Some patients experienced multiple outcomes. The aORs for treatment failure were as follows: age (aOR = 1.01, *p* = 0.737), female sex (aOR = 0.31, *p* = 0.297), maximum abscess diameter (aOR = 1.45, *p* = 0.006), presence of diabetes (aOR = 0.16, *p* = 0.070), and presence of multilocular abscesses (aOR = 3.88, *p* = 0.030). Notably, the abscess cavity size and multilocular formation were significantly associated with an increased risk of treatment failure.

**Conclusions:**

Abscess size and multiloculation are significant prognostic factors of lung abscess treatment failure. Early detection through imaging and laboratory evaluations may aid in risk stratification and prompt clinical decision-making.

**Clinical trial registration:**

The participants were registered retrospectively.

**Supplementary Information:**

The online version contains supplementary material available at 10.1186/s12890-025-04002-3.

## Background

A lung abscess is a localized area of necrosis and pus within the lung parenchyma, forming a cavity often characterized by an air fluid level [1]. Since antibiotics were introduced, lung abscess incidence and mortality rates have declined [2]. However, a lung abscess remains a serious condition with significant mortality, reaching 15.8% at 1 year, and may require interventions like drainage or surgery [1, 3–5]. The standard treatment for lung abscesses involves broad-spectrum antibiotics, including those against anaerobic bacteria [6]. However, in some cases, antibiotic treatment is insufficient, necessitating drainage or surgical intervention [3]. Given that a certain proportion of patients fail to respond to antibiotic treatment, identifying clear prognostic factors is essential for optimizing therapeutic strategies.

To date, only limited data are available on prognostic factors associated with lung abscess [5, 7–9]. Previous single-institution prospective studies have suggested that age, male gender, smoking status, alcohol intake, diabetes mellitus, larger abscess size, and anemia may be linked to mortality [7, 8]. However, the small number of cases and reliance on univariate analyses reduce the robustness of these results. Subsequent studies using multivariate analyses also have limitations [5, 9]. For instance, a study from France was hindered by its use of a composite endpoint combining heterogeneous short- and long-term outcomes (mortality, recurrence, and radiologic sequelae), making it difficult to identify predictors specifically for critical outcomes like early mortality or the need for surgical intervention [9]. Furthermore, a study focusing only on intensive care unit patients is limited, as its findings are confined to a severely ill population [5].Taken together, the available data remain limited, and further research is warranted to comprehensively evaluate prognostic factors in patients with lung abscesses.

Therefore, we conducted a 20-year retrospective, single-center observational study of patients with lung abscess at a regional community hospital in Japan. The objective of this study was to identify prognostic factors for a composite outcome comprising in-hospital death, surgical intervention, or CT-guided drainage of the lung abscess using multivariable analysis.

## Methods

### Study setting and population

We conducted a single-center retrospective observational study of adult patients clinically diagnosed with lung abscesses who received initial treatment with conservative medical therapy. The index date was defined as the date of the lung abscess diagnosis. Patients diagnosed with a lung abscess at Kameda Medical Center between April 1, 2004, and June 30, 2024, were included. The study protocol was approved by the Institutional Review Board of the Kameda Medical Center (#24–045). Owing to the retrospective nature of the study, patient data were anonymized, and informed consent was waived.

Based on previously established diagnostic criteria [6, 10–12], lung abscess was diagnosed if all the following criteria were met: (1) imaging findings: chest X-ray and computed tomography (CT) revealing findings suggestive of lung abscess (on CT, suggestive findings were defined as any of the following: (a) a thick-walled cavity ≥ 1 cm in diameter; (b) an intralesional air–fluid level; (c) multiloculation, defined as ≥ 2 internal septa visible on contiguous axial slices; or (d) surrounding consolidation with central low-attenuation area); (2) clinical symptoms: presence of at least two of the following symptoms: cough, dyspnea, chest pain, dullness on percussion, adventitious sounds on auscultation, or purulent sputum; (3) inflammatory findings: presence of at least one of the following: elevated white blood cell count (≥ 10,000/µL), leukopenia (≤ 4,000/µL), or left shift in differential count.

The exclusion criteria were as follows: (1) patients who did not receive treatment for lung abscess; (2) known cases of cavitary lung cancer.

### Treatment strategy

All patients diagnosed with lung abscess received antibiotic therapy, primarily consisting of agents with anaerobic coverage [1, 2, 13]. If clinical deterioration or radiological worsening (expansion of the abscess) was observed within 1–2 weeks of treatment initiation, surgical treatment or CT-guided drainage was considered [1–3, 13, 14].

### Outcomes

The primary outcome was the association between patient characteristics at the time of lung abscess diagnosis and treatment failure. Treatment failure was defined as a composite outcome including surgical intervention, CT-guided drainage of the lung abscess, or in-hospital death.

### Data collection

We retrospectively collected comprehensive data from each patient’s medical records. This information included patient demographics (age, sex, Eastern Cooperative Oncology Group Performance Status [ECOG PS]) and lifestyle history, with smoking status categorized as “none,” “past,” or “current,” and daily alcohol intake classified as “none,” “< 60 g/day,” or “≥ 60 g/day.” We documented medical history, including prior lung illnesses (such as chronic obstructive pulmonary disease and asthma), a history of aspiration pneumonia, and other comorbidities like malignancy and diabetes mellitus, with the overall burden of comorbidity evaluated using the Charlson Comorbidity Index. We also recorded whether patients were receiving chemotherapy or immunosuppressive therapy at time of diagnosis. The time from symptom onset to hospital admission was also recorded. Laboratory data at diagnosis, including hematology and biochemistry, and microbiological results (sputum, pleural fluid, blood, drainage aspirate cultures) were collected. For sputum culture results, we defined culture positivity as the detection of organisms with a Geckler classification of 3–5 on Gram stain and those generally considered potential pathogens of pneumonia or lung abscess [15–17]. Given the aspiration-related pathogenesis of lung abscess [1], particularly in patients with poor ECOG PS [18], we accepted Geckler class 3–5 sputum as evaluable for etiologic assessment [19, 20]. A detailed review of chest CT scans was performed to assess imaging characteristics. These included the abscess location, maximum diameter, cavity formation, presence of multiloculation (defined as ≥ 2 internal septa visible on contiguous axial slices), pleural effusion, lung abscess-related empyema, emphysema, and the presence of unilateral or bilateral multiple lesions. For the primary analysis of this study, two key variables—the maximum abscess diameter and the presence of multiloculation—were specifically evaluated by two pulmonologists, and the final assessment for these two items was reached by consensus. Finally, we recorded treatment details, including the antibiotic regimens used and any surgical or drainage procedures performed.

### Statistical analysis

As this was a retrospective observational study, no sample size calculation was performed, and all available cases were included. For statistical analysis, variables with missing data were handled as follows. Data were missing for smoking history (*n* = 2), alcohol intake (*n* = 5), and neutrophil count (*n* = 3); analyses involving these variables were conducted using the available cases without imputation. For microbiological evaluation, sputum, pleural fluid, blood, and drainage aspirate cultures that were not obtained were classified as negative. Patient characteristics were compared between the treatment success and failure groups. Continuous variables were analyzed using the t-test, whereas categorical variables were analyzed using the chi-square test. Statistical significance was set at *p* < 0.05. To identify predictors of treatment failure, we developed a logistic regression model. The selection of covariates for adjustment was necessarily limited by the small number of events, so we prioritized variables based on previous studies and clinical relevance, selecting age, sex, diabetes, and abscess characteristics (diameter and loculation) [5, 7–9]. Statistical analyses were performed using R (version 4.1.0; R Core Team, 2021).

## Results

**Fig. 1:**
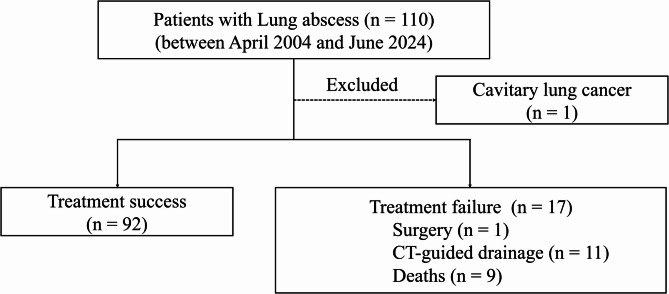
Flowchart of patient selection. In the treatment failure group, some patients met multiple endpoints, including surgery, CT-guided drainage, and death. CT, computed tomography

A total of 109 patients diagnosed with lung abscesses between April 2004 and June 2024 met the study criteria after excluding patients with cavitary lung cancer. The mean age of the cohort was 74.0 years, and 21 patients (19.2%) were female. The patient selection process is illustrated in Fig. 1. Treatment failure was observed in 17 (15.6%) patients, including 9 (8.3%) in-hospital deaths, 1 (0.9%) surgical intervention, and 11 (10.1%) cases requiring CT-guided drainage of the lung abscess, with some patients experiencing multiple outcomes.

Table 1 shows the clinical characteristics of patients with lung abscesses. Comparison between the two groups revealed mean ages of 70.77 and 73.82 years, respectively (*p* = 0.394), with 20 and 1 female patients, respectively (*p* = 0.235). The proportion of non-smokers was 25 (27.5%) and 4 (25.0%) (*p* = 0.966), while daily alcohol consumption of ≥ 60 g was observed in 8 (9.1%) and 0 (0.0%) patients (*p* = 0.027). There was no significant difference in ECOG PS between the groups (*p* = 0.382), with 71 patients (77.2%) in the success group and 12 (70.6%) in the failure group having a PS of 0. Regarding comorbidities, 10 (10.9%) and 1 (5.9%) patients had COPD (*p* = 0.850), 22 (23.9%) and 2 (11.8%) had diabetes mellitus (*p* = 0.428), 9 (9.8%) and 3 (17.6%) had lung cancer (*p* = 0.596), and 21 (22.8%) and 2 (11.8%) had non-lung malignancies (*p* = 0.482). The mean Charlson comorbidity index was 4.83 and 4.94 (*p* = 0.845). Time from onset to admission was within 1 week in 39 (42.4%) and 6 (35.3%) patients, within 1–2 weeks in 22 (23.9%) and 0 (0.0%) patients, and more than 2 weeks in 31 (33.7%) and 11 (64.7%) patients, respectively (*p* = 0.020). Laboratory findings showed white blood cell counts of 14,092/µL and 13,852/µL (*p* = 0.887), C-reactive protein levels of 15.67 mg/dL and 19.69 mg/dL (*p* = 0.111), serum albumin levels of 2.75 g/dL and 2.23 g/dL (*p* = 0.002), hemoglobin levels of 11.88 g/dL and 10.56 g/dL (*p* = 0.008). Contrast-enhanced CT was performed in 76 (82.6%) and 12 (70.6%) patients in the treatment success and failure groups, respectively (*p* = 0.412). Chest CT findings revealed pleural effusion in 36 (39.1%) and 9 (52.9%) patients (*p* = 0.427), a mean maximum abscess diameter of 3.65 cm and 5.77 cm (*p* < 0.001), right lower lobe involvement in 16 (17.4%) and 4 (23.5%) patients (*p* = 0.795), cavity formation in 48 (52.2%) and 12 (70.6%) patients (*p* = 0.256), and multiloculated abscesses in 24 (26.1%) and 11 (64.7%) patients (*p* = 0.004). Regarding drainage of pleural effusion or empyema, manual chest drainage was performed in 10 (10.9%) patients and 1 (5.9%) patient (*p* = 0.850), respectively, and CT-guided chest drainage was performed in 7 (7.6%) and 3 (17.6%) patients (*p* = 0.390), respectively. The empiric antibiotics used are listed in Supplemental Table 1. Overall, ampicillin/sulbactam alone was the most frequently used (*n* = 67), followed by tazobactam/piperacillin alone (*n* = 10).

**Table 1:** Clinical characteristics of 109 patients with lung abscesses

Characteristics and risk factors	Treatment success	Treatment failure	*P* value
	(*n* = 92)	(*n* = 17)	
Age, mean years ± SD	70.77 ± 14.16	73.82 ± 8.88	0.394
Female	20 (21.7)	1 (5.9)	0.235
Smoking history *			
None	25 (27.5)	4 (25.0)	0.966
Past	48 (52.7)	9 (56.2)	
Current	18 (19.8)	3 (18.8)	
Alcohol intake *			
None	50 (56.8)	5 (31.2)	0.027
< 60 g/day	30 (34.1)	11 (68.8)	
≥ 60 g/day	8 (9.1)	0 (0)	
ECOG performance status			0.382
0	71 (77.2)	12 (70.6)	
1	13 (14.1)	2 (11.8)	
2	4 (4.3)	1 (5.9)	
3	2 (2.2)	0 (0.0)	
4	2 (2.2)	2 (11.8)	
Underlying lung diseases			
COPD	10 (10.9)	1 (5.9)	0.850
Asthma	7 (7.6)	0 (0)	0.524
History of aspiration pneumonia	5 (5.4)	1 (5.9)	1.000
Lung cancer	9 (9.8)	3 (17.6)	0.596
Diabetes mellitus	22 (23.9)	2 (11.8)	0.428
Extrapulmonary condition			
GERD	6 (6.5)	3 (17.6)	0.293
Malignant tumors (non-lung cancer)	21 (22.8)	2 (11.8)	0.482
Cerebrovascular disease	13 (14.1)	3 (17.6)	0.997
Liver disease	10 (10.9)	0 (0.0)	0.333
Congestive heart failure	6 (6.5)	0 (0.0)	0.614
Chronic renal disease	2 (2.2)	1 (5.9)	0.959
Renal replacement therapy	0 (0.0)	0 (0.0)	− †
Charlson comorbidity index	4.83 ± 2.22	4.94 ± 2.25	0.845
Chemotherapy or immunosuppressive therapy			
Immunosuppressive agents	2 (2.2)	0 (0.0)	1.000
Anticancer therapy	5 (5.4)	1 (5.9)	1.000
Time from onset to admission			0.020
<1 week	39 (42.4)	6 (35.3)	
1–2 weeks	22 (23.9)	0 (0.0)	
>2 weeks	31 (33.7)	11 (64.7)	
Labo data			
White blood cell count	14,092.48 ± 6,058.34	13,852.94 ± 4,615.21	0.877
Neutrophil count *	12,898.89 ± 11,968.90	11,452.93 ± 4,637.93	0.646
C-reactive protein	15.67 ± 9.48	19.69 ± 9.52	0.111
Hemoglobin (g/dl) ± SD	11.88 ± 1.86	10.56 ± 1.66	0.008
Albumin level (g/dl) ± SD	2.75 ± 0.62	2.23 ± 0.58	0.002
Maximum diameter of the lung abscess, mean cm ± SD	3.65 ± 1.92	5.77 ± 2.76	< 0.001
Lung abscess location ‡			
Right upper lobe	35 (38.0)	5 (29.4)	0.686
Right middle lobe	23 (25.0)	6 (35.3)	0.559
Right lower lobe	16 (17.4)	4 (23.5)	0.795
Left upper lobe	12 (13.0)	5 (29.4)	0.179
Left lower lobe	23 (25.0)	6 (35.3)	0.559
Pleural effusion	36 (39.1)	9 (52.9)	0.427
Lung abscess-related empyema	13 (14.1)	3 (17.6)	0.997
Emphysema	33 (35.9)	7 (41.2)	0.886
Unilateral pulmonary multiple lesions	26 (28.3)	7 (41.2)	0.437
Bilateral pulmonary multiple lesions	2 (2.2)	1 (5.9)	0.959
Cavity formation of lung abscess	48 (52.2)	12 (70.6)	0.256
Multilocular abscesses	24 (26.1)	11 (64.7)	0.004

*COPD* Chronic obstructive pulmonary disease, *ECOG* Eastern Cooperative Oncology Group, *GERD* Gastroesophageal reflux disease, *SD* standard deviation

*Data are missing for smoking history (*n* = 2), alcohol intake (*n* = 5), and neutrophil count (*n* = 3)

† Because there were no cases of renal replacement therapy in either group, the chi-square test could not be performed

‡Some patients had abscesses in multiple locations; thus, the sum of locations exceeds the number of patients

Supplemental Table 2 summarizes the microbiological characteristics of the study population. Among sputum cultures, *Klebsiella pneumoniae* was the most frequently isolated organism. Positive pleural fluid cultures were observed in 3.3% and 5.9% of the treatment success and failure groups, respectively. Positive blood cultures were detected in 5.4% and 5.9% of patients in the treatment success and failure groups, respectively. Lung abscess aspirate cultures were positive in 0% and 5.9% of the treatment success and failure groups, respectively.

Supplemental Table 3 presents the characteristics of patients who underwent CT-guided drainage of lung abscesses, among whom 4 (36.4%) patients died. The mean maximum diameter of the abscesses was 5.73 cm, and multilocular abscesses were observed in 72.7% of cases. Supplemental Table 4 shows the baseline characteristics and outcomes of patients who experienced in-hospital death. The mean maximum abscess diameter was 5.01 cm, and multilocular abscesses were observed in 66.7%. Among these patients, 11.1% had a ECOG PS of 2 and 22.2% had a ECOG PS of 4. Supplemental Table 5 provides detailed information on 11 patients who underwent CT-guided drainage of lung abscesses. In all cases, the abscesses were contiguous with the chest wall.

Table 2 shows the results of the logistic regression analysis of prognostic factors. Univariate analysis was conducted to evaluate prognostic factors for lung abscesses, including age, sex, maximum abscess diameter, presence of diabetes mellitus, and multiloculation. In this analysis, a larger maximum abscess diameter (OR = 1.46, 95% CI 1.16–1.83, *p* = 0.001) and the presence of multiloculation (OR = 5.19, 95% CI 1.73–15.58, *p* = 0.003) were significantly associated with treatment failure. Multivariate logistic regression analysis confirmed similar trends for age (adjusted odds ratios [aOR] = 1.01, 95% CI 0.96–1.06, *p* = 0.737), female sex (aOR = 0.31, 95% CI 0.03–2.79, *p* = 0.297), maximum abscess diameter (aOR = 1.45, 95% CI 1.11–1.90, *p* = 0.006), diabetes (aOR = 0.16, 95% CI 0.02–1.15, *p* = 0.070), and multiloculation (aOR = 3.88, 95% CI 1.14–13.24, *p* = 0.030) (Table 2). Notably, the abscess cavity size and multilocular formation were significantly associated with an increased risk of treatment failure.

**Table 2:** Logistic regression analysis of clinical and chest CT imaging risk factors

Factors	OR (95% CI)	*P* value	aOR (95% CI)	*P* value
Age	1.02 (0.98–1.06)	0.392	1.01 (0.96–1.06)	0.737
Sex (female)	0.23 (0.03–1.80)	0.160	0.31 (0.03–2.79)	0.297
Maximum diameter of the lung abscess	1.46 (0.16–1.83)	0.001	1.45 (1.11–1.90)	0.006
Diabetes mellitus	0.42 (0.09–2.00)	0.279	0.16 (0.02–1.15)	0.070
Multiloculation	5.19 (1.73–15.58)	0.003	3.88 (1.14–13.24)	0.030

*OR* odds ratio, *aOR* adjusted odds ratio, *CI* confidence interval

## Discussion

Our study analyzed 109 patients with lung abscesses and compared 92 cases of treatment success with 17 cases of treatment failure (requiring surgical intervention, CT-guided drainage, or in-hospital death). Among treatment failure cases, larger abscesses and multiloculated abscesses were more frequently observed. Multivariate logistic regression analysis, including age, sex, presence of diabetes mellitus, abscess cavity diameter, and multiloculated abscesses, revealed that both the abscess cavity diameter and multiloculation were significantly associated with treatment failure. In addition to cavitary diameter, the finding that multiloculation is associated with treatment failure is a novel observation.

Previous studies conducted before the year 2000 reported in-hospital mortality as high as 20%–28% [1, 7, 21]. Since then, advances in diagnostic and therapeutic approaches have markedly improved the outcomes. For instance, a recent multicenter study showed all-cause mortality at 1, 3, and 12 months post-diagnosis to be 2.7%, 7.7%, and 15.8%, respectively [4], whereas the rate for patients admitted to the ICU has been reported to be as high as 20% [5]. In this context, our observed in-hospital mortality rate of 8.3% aligns well with modern reported rates (2.7%–14.4%) [4, 6, 9], reflecting the substantial decline in mortality over the past two decades. This improvement likely stems from decades of progress in clinical management, including the routine use of CT for rapid diagnosis, the availability of potent broad-spectrum antibiotics, and the refinement of interventional drainage for refractory cases [1, 2]. However, our finding underscores that a notable portion of patients still succumb to the disease. This persistent mortality highlights the critical need to identify prognostic factors that can help guide more intensive or tailored therapies for high-risk individuals.

Several prognostic factors for lung abscess have been previously reported, including abscess size, age, male gender, diabetes mellitus, and alcohol intake [5, 7–9]. Patients with multiple risk factors tend to have worse prognoses [7]. However, these studies have limitations, as their findings are based on univariate analyses, include a mixture of short- and long-term outcomes, or are restricted to patients admitted to the ICU. We evaluated prognostic factors for a composite outcome of in-hospital death, the need for surgical intervention, and CT-guided drainage using multivariable analysis, and found that abscess size and multiloculation were significant predictors.

Regarding the impact of abscess size on prognosis, Hirshberg et al. demonstrated that larger abscess volumes and sizes were associated with worse outcomes [7]. Historically and based on clinical experience, lung abscesses measuring ≥ 6 cm in diameter have frequently been reported to be refractory to antibiotic therapy alone and often require drainage procedures [14]. Consistent with these findings, the present study confirmed that larger abscess size was significantly associated with treatment failure, indicating its role as a prognostic factor. A large abscess cavity diameter is thought to contribute to poor penetration of antimicrobial agents [22], making the infection difficult to control and increasing the need for drainage. Furthermore, if drainage is challenging, the likelihood of requiring surgical intervention also increases. A large cavity may also promote the extension of the infection into the pleural space, leading to the development of empyema [23].

Multiloculation is a characteristic chest CT finding of a lung abscess. However, few studies have statistically evaluated its clinical significance. A previous study reported multiloculated lung abscesses in 34 of 101 cases (33.7%) [23]. In our study, 35 of 109 patients (32.1%) exhibited multiloculation, a comparable proportion. Notably, multiloculated abscesses were significantly more common in the treatment failure group, demonstrating an association with poor outcomes. The pathophysiology of multiloculated lung abscesses is not well understood, and no prior studies have directly addressed this condition. Multiloculation is seen not only in empyema but also in other abscesses, such as liver abscesses. In empyema, loculated pleural effusion is a known prognostic factor [24]. The disease progression of empyema is classified into three stages: exudative, transitional fibrinopurulent, and organizing [25]. In the transitional fibrinopurulent stage, it is thought that loculation occurs as a result of increased neutrophil infiltration and fibrin deposition due to infection [25, 26]. These structural changes hinder drainage, and are associated with poor outcomes [24]. Similar to other types of abscesses, percutaneous drainage of multiple large or loculated lesions may be difficult in pyogenic liver abscesses and surgical intervention may be considered [27]. While pus in the lungs can drain spontaneously into the airways, the structure of multiloculated lung abscesses is still similar to that of empyema and pyogenic liver abscesses. Therefore, these diseases may share similar pathophysiological mechanisms [24, 27]. Further research is needed to explore the pathophysiology in greater detail; however, this study suggests that multiloculation is associated with treatment failure.

While several demographic and clinical predictors for lung abscess have been reported, our study highlights the particular importance of objective imaging features identifiable at the time of diagnosis. Specifically, a large abscess diameter and the presence of multiloculation were strongly associated with treatment failure. These findings suggest that the initial chest CT provides not only a diagnosis but also valuable prognostic information that can aid clinicians in risk stratification. Therefore, for patients identified as high-risk based on these imaging characteristics, particularly those who show an inadequate response to initial antibiotic therapy, careful monitoring and early consideration of interventional procedures, such as drainage or surgery, may be warranted to improve outcomes.

This study has some limitations. First, the single-center nature of this study limits its external validity. Additionally, the small sample size restricted our statistical power. This prevented us from including variables such as hemoglobin and albumin levels—which were significant in the univariate analysis—in the multivariate analysis due to the small number of treatment failures [7]. Furthermore, for the same reason, it was not feasible to perform separate analyses to identify risk factors for the individual components of our composite outcome, such as in-hospital death or the need for drainage, due to the limited number of events for each. To validate these findings and investigate a broader range of prognostic factors, larger, multicenter retrospective and prospective studies are essential. Second, our definition of the composite endpoint “treatment failure” included the need for drainage or surgery. However, this outcome should be interpreted with caution. The decision to perform these interventions may have been driven by baseline factors, such as a large initial abscess size, rather than the true failure of antibiotic therapy. This represents a potential indication bias. Third, the imaging assessment had limitations. Abscess size measurement relied on a subjective assessment by physicians, introducing the potential for interobserver variability. Additionally, the use of non-contrast CT in some patients may have led to an underestimation of multiloculation due to the difficulty in visualizing internal septations. Fourth, the prognostic factors we identified, abscess size and multiloculation, may serve as markers for overall illness severity and all-cause mortality risk as well as abscess-specific outcomes. This is conceptually similar to the RAPID score for pleural infection [28], where predictors can reflect systemic frailty rather than localized disease severity. However, our composite endpoint of “treatment failure” also included the need for drainage or surgery. These interventions are often driven by local, mechanical factors directly related to the abscess itself, such as poor antibiotic penetration into a large cavity or the structural complexity of a multiloculated lesion hindering drainage. Therefore, although these imaging predictors are associated with systemic risk, they are also valuable in identifying abscesses that pose specific local treatment challenges and may require early intervention. Fifth, our data were obtained from patient records, which introduced the possibility of information bias. In particular, clinical decisions regarding surgical intervention and drainage may have been influenced by the identified risk factors, leading to potential bias from the self-fulfilling prophecy.

## Conclusion

Abscess size and multiloculation were identified as potential prognostic factors of lung abscesses. Evaluating these factors at diagnosis may enable the early identification of high-risk cases and formulation of appropriate treatment strategies. Further multicenter and prospective studies are needed to validate these findings and enhance risk stratification in lung abscess management.

## Supplementary Information

Supplementary Material 1.

Supplementary Material 2.

## Data Availability

Data supporting the findings of this study are available from the corresponding author upon request. The data are not publicly available due to privacy and ethical restrictions.

## Abbreviations

OR: Odds ratio
aOR: Adjusted odds ratio
CT: Computed tomography
ECOG PS: Eastern Cooperative Oncology Group Performance Status
COPD: Chronic obstructive pulmonary disease
GERD: Gastroesophageal reflux disease
