# Supplementary material for: Prognostic factors of lung abscess: a single-center retrospective cohort study
Source: BMC Pulm Med. 2025 Nov 11;25:519. doi: 10.1186/s12890-025-04002-3 (PMC12607195; doi:10.1186/s12890-025-04002-3)
Supplement: Supplementary file 2 — Supplementary Material 2. [file 12890_2025_4002_MOESM2_ESM.docx]

**Supplementary Materials**

Supplemental Table 1. Summary of empirical antimicrobial therapy

| Antibiotic regimen | Treatment success | Treatment failure |
| --- | --- | --- |
|  | N = 92 | N = 17 |
| Ampicillin/Sulbactam | 58 (63.0) | 9 (52.9) |
| Piperacillin/Tazobactam | 8 (8.7) | 2 (11.8) |
| Amoxicillin/Clavulanate + Amoxicillin | 5 (5.4) | 1 (5.9) |
| Ceftriaxone | 5 (5.4) | 1 (5.9) |
| Cefditoren Pivoxil | 3 (3.3) | 0 (0.0) |
| Cefalexin, Doxycycline | 2 (2.2) | 0 (0.0) |
| Piperacillin/Tazobactam, Vancomycin | 1 (1.1) | 1 (5.9) |
| Amoxicillin/Clavulanate + Amoxicillin, Azithromycin | 1 (1.1) | 0 (0.0) |
| Ampicillin/Sulbactam, Azithromycin | 0 (0.0) | 1 (5.9) |
| Amoxicillin, Clindamycin | 1 (1.1) | 0 (0.0) |
| Amoxicillin, Doxycycline | 1 (1.1) | 0 (0.0) |
| Cefditoren Pivoxil, Clindamycin, | 1 (1.1) | 0 (0.0) |
| Clindamycin | 1 (1.1) | 0 (0.0) |
| Ceftriaxone, Garenoxacin | 1 (1.1) | 0 (0.0) |
| Ceftriaxone, Metronidazole | 1 (1.1) | 0 (0.0) |
| Cefotaxime | 1 (1.1) | 0 (0.0) |
| Doxycycline, Amoxicillin | 1 (1.1) | 0 (0.0) |
| Doripenem, Clindamycin | 0 (0.0) | 1 (5.9) |
| Levofloxacin, Clindamycin | 1 (1.1) | 0 (0.0) |
| Meropenem | 0 (0.0) | 1 (5.9) |

NOTE: All antimicrobial agents administered on the first day of empirical therapy are listed.

Supplemental Table 2. Microbiological characteristics of the study population

| Pathogens | Treatment success  (n = 92) | Treatment failure  (n = 17) |
| --- | --- | --- |
| Sputum culture |  |  |
| Positive patients | 22 (23.9) | 6 (35.3) |
| Isolated organisms * |  |  |
| *Klebsiella pneumoniae* | 4 (4.3) | 2 (11.8) |
| *Methicillin-susceptible Staphylococcus aureus* | 3 (3.3) | 0 (0.0) |
| *Haemophilus parainfluenzae* | 1 (1.1) | 2 (11.8) |
| *Haemophilus influenzae* | 2 (2.2) | 0 (0.0) |
| *Escherichia coli* | 2 (2.2) | 0 (0.0) |
| *Klebsiella oxytoca* | 2 (2.2) | 0 (0.0) |
| *Methicillin-resistant Staphylococcus aureus* | 1 (1.1) | 1 (5.9) |
| *Streptococcus constellatus* | 2 (2.2) | 0 (0.0) |
| *Streptococcus pneumoniae* | 2 (2.2) | 0 (0.0) |
| *Citrobacter koseri* | 1 (1.1) | 0 (0.0) |
| *Enterobacter aerogenes* | 1 (1.1) | 0 (0.0) |
| *Haemophilus* spp. | 1 (1.1) | 0 (0.0) |
| *Prevotella intermedia* | 1 (1.1) | 0 (0.0) |
| *Pseudomonas aeruginosa* | 1 (1.1) | 0 (0.0) |
| *Streptococcus intermedius* | 1 (1.1) | 0 (0.0) |
| *Fusobacterium nucleatum* | 0 (0.0) | 1 (5.9) |
| *Streptococcus milleri group* | 0 (0.0) | 1 (5.9) |
| Pleural fluid culture |  |  |
| Positive patients * | 3 (3.3) | 1 (5.9) |
| Isolated organisms |  |  |
| *Peptostreptococcus sp.* | 1 (1.1) | 0 (0.0) |
| *Streptococcus milleri* | 0 (0.0) | 1 (5.9) |
| *Streptococcus intermedius* | 1 (1.1) | 0 (0.0) |
| *Streptococcus constellatus* | 1 (1.1) | 0 (0.0) |
| *Prevotella intermedia* | 1 (1.1) | 0 (0.0) |
| Blood culture |  |  |
| Positive patients | 5 (5.4) | 1 (5.9) |
| Isolated organisms |  |  |
| *Staphylococcus aureus* | 2 (2.2) | 0 (0.0) |
| *Streptococcus pneumoniae* | 1 (1.1) | 0 (0.0) |
| *viridans streptococcus* | 1 (1.1) | 0 (0.0) |
| *Streptococcus constellatus* | 1 (1.1) | 0 (0.0) |
| *Staphylococcus ureolyticus* | 0 (0.0) | 1 (5.9) |
| Lung abscess aspirate culture |  |  |
| Positive patients | 0 (0.0) | 1 (5.9) |
| Isolated organisms |  |  |
| *Streptococcus constellatus* | 0 (0.0) | 1 (5.9) |

*Multiple organisms were isolated from some patients; thus, the sum of the individual organisms exceeds the number of positive patients

Supplemental Table 3 Baseline characteristics and outcomes of patients who underwent CT-guided drainage of the lung abscess

| Characteristics and risk factors | CT-guided drainage of the lung abscess |
| --- | --- |
|  | (n = 11) |
| Age, mean years ± SD | 73.73 ± 9.38 |
| Female | 0 (0.0) |
| Smoking history |  |
| None | 2 (18.2) |
| Past | 7 (63.6) |
| Current | 2 (18.2) |
| Alcohol intake |  |
| None | 2 (18.2) |
| < 60 g/day | 9 (81.8) |
| ≥ 60 g/day | 0 (0.0) |
| ECOG performance status |  |
| 0 | 11 (100.0) |
| 1 | 0 (0.0) |
| 2 | 0 (0.0) |
| 3 | 0 (0.0) |
| 4 | 0 (0.0) |
| Underlying lung diseases |  |
| COPD | 1 (9.1) |
| Asthma | 0 (0.0) |
| History of aspiration pneumonia | 0 (0.0) |
| Lung cancer | 1 (9.1) |
| Extrapulmonary condition |  |
| Diabetes mellitus | 1 (9.1) |
| GERD | 3 (27.3) |
| Malignant tumors (non-lung cancer) | 1 (9.1) |
| Cerebrovascular disease | 3 (27.3) |
| Liver disease | 0 (0.0) |
| Congestive heart failure | 0 (0.0) |
| Renal disease | 0 (0.0) |
| Dialysis | 0 (0.0) |
| Charlson comorbidity index | 4.91 ± 2.55 |
| Chemotherapy or immunosuppressive therapy |  |
| Immunosuppressive agents | 0 (0.0) |
| Anticancer therapy | 1 (9.1) |
| Time from onset to admission |  |
| <1 week | 3 (27.3) |
| 1~2 weeks | 0 (0.0) |
| >2 weeks | 8 (72.7) |
| Labo data |  |
| White blood cell count (/μL) ± SD | 13700.00 ± 4896.33 |
| Neutrophil count (/μL) ± SD * | 10899.06 ± 4705.05 |
| C-reactive protein (mg/dL) ± SD | 17.91 ± 10.75 |
| Hemoglobin (g/dl) ± SD | 10.97 ± 1.45 |
| Albumin level (g/dl) ± SD | 2.37 ± 0.53 |
| Maximum diameter of the lung abscess, mean cm ± SD | 5.73 ± 2.73 |
| Lung abscess location † |  |
| Right upper lobe | 3 (27.3) |
| Right middle lobe | 3 (27.3) |
| Right lower lobe | 2 (18.2) |
| Left upper lobe | 4 (36.4) |
| Left lower lobe | 4 (36.4) |
| Pleural effusion | 7 (63.6) |
| Lung abscess-related empyema | 2 (18.2) |
| Emphysema | 6 (54.5) |
| Unilateral pulmonary multiple lesions | 4 (36.4) |
| Bilateral pulmonary multiple lesions | 0 (0.0) |
| Cavity formation of lung abscess | 6 (54.5) |
| Multilocular abscesses | 8 (72.7) |
| Drainage for pleural effusion or empyema |  |
| Manual chest drainage | 0 (0.0) |
| CT-guided chest drainage | 1 (9.1) |
| In-hospital death | 4 (36.4) |

* Neutrophil count data are missing (n=1).

† Some patients had abscesses in multiple locations; thus, the sum of locations exceeds the number of patients.

COPD, Chronic obstructive pulmonary disease; CT, computed tomography; ECOG, Eastern Cooperative Oncology Group; GERD, Gastroesophageal reflux disease; SD, standard deviation

Supplemental Table 4 Baseline characteristics and outcomes of patients who experienced in-hospital death

| Characteristics and risk factors | In-hospital death |
| --- | --- |
|  | (n = 9) |
| Age, mean years ± SD | 77.22 ± 7.79 |
| Female | 1 (11.1) |
| Smoking history * |  |
| None | 2 (25.0) |
| Past | 4 (50.0) |
| Current | 2 (25.0) |
| Alcohol intake * |  |
| None | 3 (37.5) |
| < 60 g/day | 5 (62.5) |
| ≥ 60 g/day | 0 (0.0) |
| Pre-morbid ECOG performance status |  |
| 0 | 4 (44.4) |
| 1 | 2 (22.2) |
| 2 | 1 (11.1) |
| 3 | 0 (0.0) |
| 4 | 2 (22.2) |
| Underlying lung diseases |  |
| COPD | 0 (0.0) |
| Asthma | 0 (0.0) |
| History of aspiration pneumonia | 1 (11.1) |
| Lung cancer | 3 (33.3) |
| Extrapulmonary condition |  |
| Diabetes mellitus | 0 (0.0) |
| GERD | 1 (11.1) |
| Malignant tumors (non-lung cancer) | 1 (11.1) |
| Cerebrovascular disease | 1 (11.1) |
| Liver disease | 0 (0.0) |
| Congestive heart failure | 0 (0.0) |
| Renal disease | 1 (11.1) |
| Dialysis | 0 (0.0) |
| Charlson comorbidity index | 5.22 ± 2.68 |
| Chemotherapy or immunosuppressive therapy |  |
| Immunosuppressive agents | 0 (0.0) |
| Anticancer therapy | 0 (0.0) |
| Time from onset to admission |  |
| <1 week | 4 (44.4) |
| 1~2 weeks | 0 (0.0) |
| >2 weeks | 5 (55.6) |
| Labo data |  |
| White blood cell count (/μL) ± SD | 14633.33 ± 5363.30 |
| Neutrophil count (/μL) ± SD * | 12219.30 ± 4918.81 |
| C-reactive protein (mg/dL) ± SD | 20.62 ± 9.85 |
| Hemoglobin (g/dl) ± SD | 10.51 ± 1.43 |
| Albumin level (g/dl) ± SD | 2.18 ± 0.65 |
| Maximum diameter of the lung abscess, mean cm ± SD | 5.01 ± 1.10 |
| Lung abscess location † |  |
| Right upper lobe | 3 (33.3) |
| Right middle lobe | 4 (44.4) |
| Right lower lobe | 2 (22.2) |
| Left upper lobe | 2 (22.2) |
| Left lower lobe | 4 (44.4) |
| Pleural effusion | 5 (55.6) |
| Lung abscess-related empyema | 2 (22.2) |
| Emphysema | 2 (22.2) |
| Unilateral pulmonary multiple lesions | 5 (55.6) |
| Bilateral pulmonary multiple lesions | 1 (11.1) |
| Cavity formation of lung abscess | 8 (88.9) |
| Multilocular abscesses | 6 (66.7) |
| Drainage for pleural effusion or empyema |  |
| Manual chest drainage | 1 (11.1) |
| CT-guided chest drainage | 3 (33.3) |
| CT-guided drainage of the lung abscess | 4 (44.4) |

* Data are missing for smoking history (n=1), alcohol intake (n=1), and neutrophil count (n=1)

† Some patients had abscesses in multiple locations; thus, the sum of locations exceeds the number of patients.

COPD, Chronic obstructive pulmonary disease; CT, computed tomography; ECOG, Eastern Cooperative Oncology Group; GERD, Gastroesophageal reflux disease; SD, standard deviation

| Patient | Age | Sex | WBC (/µL) | CRP (mg/dL) | Albumin (g/dl) | Maximum abscess diameter (cm) | Multilocular abscesses | Distance between the abscess and the chest wall (mm) | Pleural effusion | Drainage for pleural effusion | Outcome |
| --- | --- | --- | --- | --- | --- | --- | --- | --- | --- | --- | --- |
| 1 | 70s | Male | 20600 | 9.54 | 2.1 | 5.6 | Present | 0 (adjacent) | Present | Yes | Death |
| 2 | 90s | Male | 14900 | 7.14 | 2.1 | 5.7 | Present | 0 (adjacent) | Present | No | Death |
| 3 | 70s | Male | 6200 | 39.62 | 2.4 | 6.05 | Present | 0 (adjacent) | Absent | No | Death |
| 4 | 70s | Male | 17200 | 24.12 | 2.4 | 5.7 | Present | 0 (adjacent) | Present | No | Recovery |
| 5 | 70s | Male | 12900 | 24.73 | 1.3 | 10.3 | Present | 0 (adjacent) | Absent | No | Recovery |
| 6 | 80s | Male | 18000 | 13.8 | 2.3 | 5.19 | Absent | 0 (adjacent) | Present | No | Recovery |
| 7 | 60s | Male | 8100 | 14.55 | 2.6 | 1.92 | Absent | 0 (adjacent) | Present | No | Recovery |
| 8 | 70s | Male | 9000 | 5.07 | 3.2 | 3.32 | Present | 0 (adjacent) | Absent | No | Recovery |
| 9 | 60s | Male | 14900 | 27.4 | 2.1 | 11 | Absent | 0 (adjacent) | Present | No | Recovery |
| 10 | 50s | Male | 9600 | 7.79 | 2.4 | 3.86 | Present | 0 (adjacent) | Absent | No | Recovery |
| 11 | 70s | Male | 19300 | 23.25 | 3.2 | 4.35 | Present | 0 (adjacent) | Present | No | Death |

Supplemental Table 5 Detailed characteristics and outcomes of 11 patients who underwent CT-guided drainage of the lung abscess

CT, computed tomography; WBC, white blood cell count; CRP, C-reactive protein
